# Association of family history with patient characteristics and prognosis in a large European gastroesophageal cancer cohort

**DOI:** 10.1007/s00508-024-02432-3

**Published:** 2024-09-05

**Authors:** Hannah C. Puhr, Luzia Berchtold, Linda Zingerle, Melanie Felfernig, Lisa Weissenbacher, Gerd Jomrich, Reza Asari, Sebastian F. Schoppmann, Gerald W. Prager, Elisabeth S. Bergen, Anna S. Berghoff, Matthias Preusser, Aysegül Ilhan-Mutlu

**Affiliations:** 1 Department of Medicine I, Division of Oncology, Medical University of Vienna, Waehringer Guertel 18–20, 1090 Vienna, Austria; 2 Institute for Medical Statistics, Center for Medical Data Science, Medical University of Vienna, Vienna, Austria; 3 Department of Surgery, Medical University of Vienna, Waehringer Guertel 18–20, 1090 Vienna, Austria

**Keywords:** Gastric cancer, Esophageal cancer, Genetics, Epidemiology, Hereditary

## Abstract

**Introduction:**

The role of the family history in the development and prognosis of gastroesophageal cancer is a controversially discussed topic as appropriate data from western cohorts are lacking. This study aims to explore its associations with disease and outcome parameters in a large European cohort.

**Methods:**

We retrospectively analyzed self-reported family history in patients with gastroesophageal cancer treated between 1 January 1990 and 31 December 2021 at the Medical University of Vienna. Association analyses with patient characteristics, tumor characteristics, symptoms and overall survival (OS) were performed.

**Results:**

In our cohort of 1762 gastroesophageal cancer patients, 592 (34%) reported a positive family history of cancer (159, 9%, gastroesophageal cancer). No associations were found with histopathological parameters or initial symptoms; however, a positive family history correlated with female gender (cancer in general: *p* = 0.011; gastroesophageal cancer: *p* = 0.015). Family history of cancer in general was associated with earlier cancer stages (*p* = 0.04), higher BMI (*p* = 0.005), and alcohol consumption (*p* = 0.010), while a positive history for gastroesophageal cancer was associated with higher age at diagnosis (*p* = 0.002) and stomach cancer (*p* = 0.002). There was no statistically significant association of positive family history with OS (*p* = 0.1, *p* = 0.45), also not in subgroups for histology (adeno and squamous cell), number of family members and degree of relative.

**Conclusion:**

Our results emphasize that a positive family history is neither statistically significantly associated with prognosis nor with specific histopathological features in patients with gastroesophageal cancer. Yet, associations with distinct patient characteristics and positive family history indicate that specific subgroups might profit from endoscopic surveillance. Prospective studies are warranted to investigate these findings further.

**Supplementary Information:**

The online version of this article (10.1007/s00508-024-02432-3) contains supplementary material, which is available to authorized users.

## Introduction

Gastroesophageal cancer is a devastating disease with high mortality rates worldwide particularly in younger patients [1]. Moreover, the longstanding belief that the prevalence is highest in Asian patients due to lifestyle and environmental factors has to be challenged as Asian expats in northern America still show higher rates than Caucasian whites (sic) [2, 3].

Thus, it is surmised that genetic factors might play an important role in gastroesophageal cancer development; however, so far only one distinguished hereditary cancer syndrome could be associated with particularly high frequency of gastroesophageal malignancies. Hereditary diffuse gastric cancer (HDGC) is an autosomal dominant syndrome widely caused by mutations in the tumor suppressor gene *CDH1* [4]. Other cancer syndromes, such as Lynch syndrome which is characterized by mismatch repair deficiency and is most commonly associated with colorectal cancer, might also lead to increased rates of upper gastrointestinal malignancies [5–7]. Yet, only a minority of gastroesophageal cancer patients are diagnosed with genetic cancer syndromes while familial accumulation is frequently observed and often believed to be associated with *Helicobacter pylori* infection, younger age at cancer onset and reduced survival time [8, 9].

Limited data exist on the impact of family history on the characteristics and prognosis of these patients. Thus, knowledge and awareness about familial gastroesophageal cancer, especially in Caucasian patients, have to be enhanced to optimize patient care and identify relatives who might profit from surveillance strategies [10, 11]. Our study focuses on examining gastroesophageal cancer patients with a self-reported family history in a western cohort, aiming to improve understanding of familial disease patterns and enhance care through potential genetic screening and increased surveillance.

## Patients, material and methods

### Patient and data recruitment

Patients aged ≥ 18 years, who were diagnosed and/or treated for pathologically confirmed gastroesophageal cancer between 1 January 1990 and 31 December 2021 at the Medical University of Vienna were included in this analysis. Programmed cell death ligand 1 (PD-L1) positivity was defined as combined positive score (CPS) ≥ 1 and/or tumor proportion score (TPS) ≥ 1 and is routinely performed at our hospital since 2020. Human epidermal growth factor receptor 2 (HER2) positivity was defined as immunohistochemical staining +++ or ++ with *HER‑2* gene amplification and has been routinely performed since 2012. Mismatch repair deficiency (dMMR) was evaluated by immunohistochemical staining and although not routinely performed is available in a subset of patients. Treatment strategies were reviewed by an interdisciplinary tumor board and patients received the best available treatment according to current treatment guidelines at the time of diagnosis.

Clinical data, including patient characteristics, tumor characteristics, symptoms, treatment strategies as well as data on family history, were retrospectively retrieved by structured electronical chart review and then collected and stored in a FilemakerPro®-based (Claris International, Santa Clara, California, USA) database. The quality of retrospective data was ensured by standardized history taking. Information on patients’ genetics was not available due to regulatory restrictions in Austria.

Family history was considered positive when a first, second or third-degree relative suffered from a patient-reported oncological disease. First-degree was classified as parents, siblings and children. Second-degree was classified as grandparents, grandchildren, aunts/uncles and nieces/nephews. Third-degree was classified as great-grandparents, great-aunts/great-uncles and first cousins [12]. If multiple relatives had a history of (gastroesophageal) cancer, the relative with the closest degree was taken into account for our analysis.

Alcohol consumption was classified as “none” in patients without any alcohol intake, “moderate” in patients with < 2 alcoholic beverages a day and “abuse” in patients with ≥ 2 alcoholic beverages a day according to international guidelines. Nicotine abuse was defined as positive when either active smoking was still ongoing and/or the patient had a history of ≥ 10 pack years.

Death was registered according to hospital chart data and/or public data provided by Statistik Austria. Patients without a registered date of death at the time of data cut-off were censored at the date that they were last known to be alive. Overall survival (OS) was defined as the time from initial cancer diagnosis to the patient’s death or last follow-up date.

All procedures were performed following the ethical standards of the responsible committee on human experimentation and with the Helsinki Declaration of 1964 and later versions. The study was approved by the ethics committee of the Medical University of Vienna (reference number: 1600/2021).

### Statistical analysis

Descriptive analyses of clinical data were conducted using measures such as the median, range and interquartile range (IQR) for continuous data. For categorical variables absolute numbers, percentages and number of missing values were reported. Differences between groups were assessed using the χ^2^-test for categorical data or Wilcoxon rank sum test for continuous data. Missing values are stated in the respective tables.

Comparisons of OS were performed using log-rank tests and Kaplan Meier estimator, which was used to estimate median OS and visualize survival curves. For testing the effect size of the parameters on OS we additionally performed Cox regression analysis. For possible confounding factors that were significant within the univariable analysis as well as in association with family history, an interaction coefficient in the Cox regression model was added.

Multivariable analysis included possible confounding factors and the interaction term if statistically significant in the bivariable model. Additionally, clinically relevant factors such as stage, tumor location and histological subtype were included. Two multivariable Cox regression models were built, one with family history in general and one with family history in the upper gastrointestinal (GI) tract.

Two-tailed *p*-values < 0.05 were considered to indicate statistical significance. Due to the hypothesis-generating design of the studies, no correction for multiple testing was applied unless specified otherwise [8]. All statistical analyses were performed using R Studio software (version 4.2.3; R Foundation for Statistical Computing, Vienna, Austria).

## Results

### Patient and tumor characteristics

Our cohort of gastroesophageal cancer patients consists of 1762 patients, who were treated at the Medical University of Vienna between 1 January 1990 and 31 December 2021. In this western cohort, 21 (1%) patients were of Asian ethnicity (3 Japan, 10 China, 2 India, 1 Kirgizstan, 1 Thailand, 1 Mongolia, 1 Vietnam, 2 Korea). Median age at first diagnosis of gastroesophageal cancer was 64 years (range 23–92 years, IQR: 55–72 years) in the overall cohort and 1235 (70%) patients were men. At the time of data cut-off (1 November 2022) 1353 (77%) patients were already deceased.

Median overall survival (OS) from cancer diagnosis to death was 21.5 months in the overall cohort (95% CI 20.6–23.1 months).

Other patient and tumor characteristics as well as symptoms at first cancer diagnosis are available in Supplementary tables 1–3. Therapeutic strategies applied in this cohort are summarized in Supplementary table 4.

### Family history in gastroesophageal cancer patients

In our cohort of 1762 gastroesophageal cancer patients 826 (58%) reported no history of malignant diseases in their family and 592 (42%) had a close relative with cancer diagnosis. Of those with positive family history in the overall cohort, 159 (27%) had known gastroesophageal cancer in their family. In patients with a positive family history in general, 496 (84%) were first-degree relatives, 63 (11%) were second-degree relatives and 3 (1%) were third-degree relatives, 387 (65%) had only one family member and 176 (30%) multiple family members with cancer diagnoses. In patients with a positive family history of gastroesophageal cancer 113 (71%) were first-degree, 38 (24%) second-degree and 3 (2%) third-degree relatives. In 30 patients the information about the degree of the relative was missing, 131 (82%) patients with a positive family history of gastroesophageal cancer had only one recorded family member and 24 (15%) had multiple family members with gastroesophageal cancer.

Of the patients with Asian ethnicity 6 (29%) patients had a positive family history of cancer in general, with 5 (83%) of them for gastroesophageal cancer.

#### Age analysis in patients with positive family history

The median age for patients with positive family history in general was 64 years (range 27–92 years, IQR: 55–71 years) compared to 63 years (range: 23–92 years, IQR: 54–72 years) in patients without a family history of cancer. The median age for patients with positive family history of gastroesophageal cancer was 66 years (range: 30–91, IQR: 57–75) compared to 63 years (range: 23–92, IQR: 54–71) in patients without family history of gastroesophageal cancer. Positive family history and age groups are shown in Supplementary figure 1. The median age for patients with a positive family history and Asian ethnicity was 47 years (range: 30–71 years), while the median age for Asian patients without positive family history was 65 years (range: 40–78 years).

#### Association with patient and tumor characteristics

Concerning the association of a positive family history with patient and tumor characteristics, the family history in general was associated with sex (*p* = 0.011), BMI (*p* = 0.040), alcohol consumption (*p* = 0.010) and stage (*p* = 0.043). A family history of gastroesophageal cancer was associated with sex (*p* = 0.015), age (*p* = 0.031) and tumor location (*p* = 0.002). Other patient and tumor parameters are listed in Table 1.

**Table 1:** Association of a family history in general and for upper gastrointestinal cancer with patient and tumor characteristics

	Family history—cancer in general			Family history—gastroesophageal cancer		
	Negative (*n* = 826)	Positive (*n* = 592)	*p* value	Negative (*n* = 1259)	Positive (*n* = 159)	*p* value
**Sex**			**0.011**	–		**0.015**
Male	595 (72%)	389 (66%)	–	887 (70%)	97 (61%)	–
Female	231 (28%)	203 (34%)		372 (30%)	62 (39%)	
**Age (years)**			**0.59**	**–**		**0.002**
Median (IQR)	63 (54–72)	64 (55–71)	–	63 (54–71)	66 (57–75)	**–**
*Age groups*			*0.65*	*–*		*0.031*
≤ 45 years	67 (8%)	50 (8%)	–	105 (8%)	12 (8%)	–
46–64 years	379 (46%)	257 (43%)		579 (46%)	57 (36%)	
≥ 65 years	380 (46%)	285 (48%)		575 (46%)	90 (57%)	
**BMI**			**0.005**	**–**		**0.16**
Median (IQR)	24.2 (21.6–27.0)	24.7 (22.2–28.1)	**–**	24.4 (21.8–27.3)	24.7 (22.2–28.1)	–
*BMI groups*			*0.040*	*–*		*0.16*
Underweight	44 (7%)	16 (3%)	–	58 (6%)	2 (2%)	–
Normal weight	355 (53%)	234 (50%)		524 (52%)	65 (51%)	
Overweight	196 (29%)	151 (32%)		308 (30%)	39 (31%)	
Obese	79 (12%)	69 (15%)		127 (12%)	21 (17%)	
Missing	152	122		242	32	
**Nicotine**			**0.88**	**–**		**0.12**
No	344 (43%)	242 (42%)	–	512 (42%)	74 (48%)	–
Abuse	463 (57%)	331 (58%)		715 (58%)	79 (52%)	
Missing	19	19		32	6	
**Alcohol**			**0.010**	**–**		**0.57**
No	393 (49%)	235 (41%)	–	552 (45%)	76 (49%)	–
Moderate	308 (38%)	262 (46%)		509 (42%)	61 (40%)	
Abuse	107 (13%)	70 (12%)		160 (13%)	17(11%)	
Missing	18	25		38	5	
**Other primary tumors**			**0.68**	**–**		**0.72**
No	650 (80%)	456 (79%)	–	984 (79%)	122 (78%)	–
Yes	166 (20%)	123 (21%)		255 (21%)	34 (22%)	
**Histological subtype**			**0.086**	**–**		**0.11**
AC	661 (80%)	495 (84%)	–	1019 (81%)	137 (86%)	–
SCC	165 (20%)	97 (16%)		240 (19%)	22 (14%)	
**Tumor location**			**0.093**	**–**		**0.002**
GEJ	250 (30%)	197 (33%)	–	394 (31%)	53 (32%)	–
Stomach	322 (39%)	244 (41%)		487 (39%)	79 (50%)	
Esphagus	254 (31%)	151 (26%)		378 (30%)	27 (17%)	
**Stage**			**0.043**	**–**		**0.37**
1	94 (11%)	95 (16%)	–	164 (13)	25 (16%)	–
2	154 (19%)	109 (18%)		233 (19%)	30 (19%)	
3	264 (32%)	193 (33%)		415 (33%)	42 (26%)	
4	314 (38%)	195 (33%)		447 (36%)	62 (39%)	
**Lauren classification**			**0.51**	**–**		**0.88**
Intestinal	167 (43%)	133 (46%)	–	257 (44%)	43 (47%)	–
Diffuse	194 (51%)	142 (49%)		292 (50%)	44 (48%)	
Mixed	23 (6%)	12 (4%)		31 (5%)	4 (4%)	
Missing	442	305		679	68	
**Signet ring cells**			**0.049**	**–**		**0.16**
No	576 (70%)	387 (65%)	–	862 (69%)	101 (64%)	–
Yes	242 (30%)	204 (35%)		388 (31%)	58 (36%)	
Missing	8	1		9	0	
***Helicobacter* infection**			**0.14**	**–**		**0.87**
Yes	230 (43%)	184 (48%)	–	366 (45%)	48 (44%)	–
No	303 (57%)	199 (52%)		442 (55%)	60 (56%)	
Missing	293	209		451	51	
**MMR**			**0.75**	**–**		**>** **0.99**
dMMR	5 (5%)	5 (6%)	–	9 (6%)	1 (6%)	–
pMMR	91 (95%)	73 (94%)		147 (94%)	17 (94%)	
Missing	730	514		1103	141	
**HER2**			**0.10**	**–**		**0.11**
Positive	66 (23%)	38 (18%)	–	96 (22%)	8 (13%)	–
Negative	215 (77%)	179 (82%)		341 (78%)	53 (87%)	
Missing	545	375		822	98	
**PD-L1**			**0.59**	**–**		**0.52**
Positive	45 (64%)	37 (60%)	–	75 (63%)	7 (54%)	–
Negative	25 (36%)	25 (40%)		44 (37%)	6 (46%)	
Missing	756	530		1140	146	

*AC* Adenocarcinoma, *BMI* Body mass index, *CPS* Combined positive score, *dMMR* Mismatch repair deficiency, *GEJ* Gastroesophageal junction, *HER2* Human epidermal growth factor receptor 2, *PD-L1* Programmed cell death ligand 1, *SCC* Squamous cell carcinoma, *TPS* Tumor proportion score

Symptoms at first diagnosis were neither associated with family history in general nor with gastroesophageal cancer and are shown in Table 2.

**Table 2:** Association of family history in general and gastroesophageal cancer with symptoms at initial diagnosis

	Family history—cancer in general			Family history—gastroesophageal cancer		
	Negative (*n* = 826)	Positive (*n* = 592)	*p* value	Negative (*n* = 1259)	Positive (*n* = 159)	*p* value
*Dysphagia*			*0.32*	*–*		*0.10*
No	314 (40%)	236 (43%)	–	481 (40%)	69 (47%)	–
Yes	475 (60%)	319 (57%)		717 (60%)	77 (53%)	
Missing	37	37		61	13	
*Dyspepsia*			*0.28*	*–*		*0.71*
No	177 (22%)	113 (20%)	–	260 (22%)	30 (20%)	–
Yes	610 (78%)	451 (80%)		943 (78%)	118 (80%)	
Missing	39	28		56	11	
*Acid reflux*			*0.65*	*–*		0.40
No	599 (84%)	427 (83%)	–	911 (84%)	115 (86%)	–
Yes	111 (16%)	85 (16%)		178 (16%)	18 (14%)	
Missing	116	80		170	26	
*Abdominal pain*			*0.97*	*–*		*0.82*
No	523 (74%)	376 (74%)	–	800 (74%)	99 (74%)	–
Yes	187 (26%)	135 (26%)		288 (26%)	34 (26%)	
Missing	116	81		171	26	
*Nausea*			0.19	–		0.60
No	595 (84%)	443 (87%)	–	923 (85%)	115 (86%)	–
Yes	115 (16%)	69 (13%)		166 (15%)	18 (14%)	
Missing	116	80		170	26	
*Weight loss*			*0.60*	*–*		*0.11*
No	368 (47%)	272 (48%)	–	560 (47%)	80 (54%)	–
Yes	417 (53%)	291 (52%)		639 (53%)	69 (46%)	
Missing	41	29		72	14	
*GI bleeding*			*0.40*	*–*		*0.80*
No	608 (78%)	422 (76%)	–	920 (78%)	110 (76%)	–
Yes—ulceration	62 (8%)	56 (10%)		103 (9%)	15 (10%)	
Yes—active bleeding	107 (14%)	77 (14%)		164 (14%)	20 (14%)	
Missing	49	37		72	14	
*Frailty*			*0.39*	*–*		*0.48*
No	661 (84%)	463 (83%)	–	998 (83%)	126 (86%)	–
Yes	122 (16%)	97 (17%)		198 (17%)	21 (14%)	
Missing	43	32		63	12	

### Overall survival (OS) analysis

**Fig. 1:**
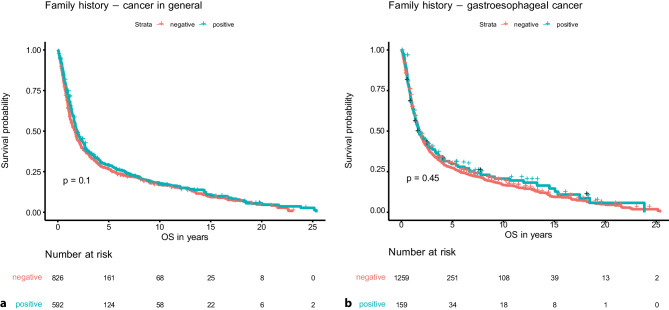
Overall survival of patients with and without positive family history of cancer in general (**a**) and gastroesophageal cancer (**b**)

Family history in general on its own was not associated with OS (negative (*n* = 826): median 20.6 months (95% CI 17.6–22.2 months); positive (*n* = 592): median 23.3 months (95% CI 20.9–26.7 months); *p* = 0.10) and neither was family history of gastroesophageal cancer (negative (*n* = 1259): median OS 21.6 months (95% CI 20.1–23.2 months); positive (*n* = 159): median 21.0 months (95% CI 16.7–32.3 months); *p* = 0.45) (see Fig. 1).

**Fig. 2:**
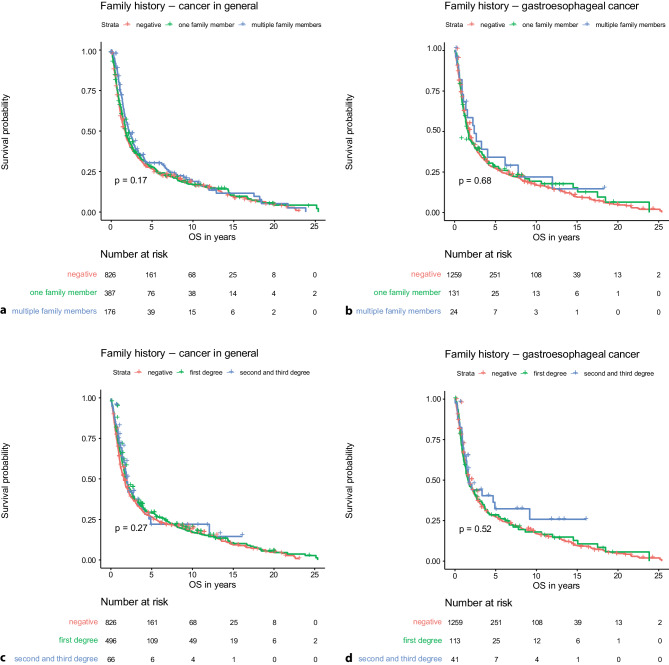
Overall survival depending on number of positive family members in patients with positive family history of cancer in general (**a**) and gastroesophageal cancer (**b**). Overall survival depending on degree of relatives with positive family history of cancer in general (**c**) and gastroesophageal cancer (**d**)

Moreover, we also analyzed whether the number of family members with a positive family history as well as the degree of relatives was associated with the OS. Both analyses showed that there is no statistically significant association, neither in patients with positive family history in general nor in patients with a positive family history of gastroesophageal cancer (negative family history vs. one family member vs. multiple family members: *p* = 0.17 and *p* = 0.68; negative family history versus positive in first-degree relatives versus positive in second or third-degree relatives: *p* = 0.27 and *p* = 0.52; respectively) (see Fig. 2).

#### Subgroup analysis for histological subtypes

In addition, subgroup analysis for histological subtypes did not show a statistically significant association with OS (family history in general: adenocarcinoma: *p* = 0.18; squamous cell carcinoma: *p* = 0.35; family history gastroesophageal cancer: adenocarcinoma: *p* = 0.78; squamous cell carcinoma: *p* = 0.97) (see Supplementary Fig. 2).

#### Subgroup analysis for age categories

When only looking at young patients with age lower or equal to 45 years at first diagnosis, neither family history in general (*p* = 0.85) nor family history of gastroesophageal cancer (*p* = 0.08) was associated with the OS either; however, for older patients aged 65 years and above, those with a family history in general showed longer OS (HR = 0.78, 95% CI 0.66–0.93; *p* = 0.005). A significant interaction between family history of gastroesophageal cancer and age on OS was observed. When family history is negative the relationship between age and OS is particularly pronounced, with longer survival for younger patients (≤ 45 vs. > 45&< 65 years: HR = 0.77, 95% CI 0.6–1, *p* = 0.047; ≥ 65 vs. > 45&< 65 years: HR = 1.14, 95% CI 1.00–1.30, *p* = 0.048); however, when the family history of gastroesophageal cancer is positive, the effect of age on OS is reversed, such that patients at age 45 years and below, had a higher risk compared to patients with age above 45 years and below 65 years (HR = 2.15, 95% CI 1.02–4.49, *p* = 0.044). Family history in general and age showed a similar interaction, where patients aged 65 years or older had better survival rates than younger patients when they had a positive family history (HR = 0.77, 95% CI 0.6–0.99, *p* = 0.042). No other significant interactions between family history and other parameters on OS were observed.

#### Subgroup analysis for stage categories

Also, family history in general and family history of gastroesophageal cancer with respect to separate tumor stages were not significantly associated with OS (stage 1: *p* = 0.64, *p* = 0.17; stage 2: *p* = 0.48, *p* = 0.93; stage 3: *p* = 0.46, *p* = 0.49; stage 4: *p* = 0.99, *p* = 0.54, respectively).

#### Multivariate survival analysis

In multivariate analysis stage (HR = 2.174, 95% CI 1.7015–2.778, *p* < 0.0001), age and tumor location stayed statistically significantly associated with OS in both models. Family history in general was significant in the multivariable model (HR = 1.37, 95% CI 1.11–1.69; *p* = 0.004), while family history for upper GI malignancies stayed insignificant (HR = 0.93, 95% CI 0.63–1.37; *p* = 0.7). Additionally, the interaction effect between family history in general and age stayed significant, while the family history of tumors in the upper GI tract and age did not. Detailed results are available in Supplementary tables 5 and 6.

## Discussion

Family history is known to be a major risk factor for diverse cancer entities [13, 14]. Public interest in hereditary cancers, such as the sustained increase in genetic testing following Angelina Jolie’s public surgery disclosure (also known as the “Angelina Jolie effect”), contrasts with the low awareness and data scarcity regarding gastroesophageal cancer familial risks [1, 15–18]. Thus, our analysis aimed to provide further insight into this subject in a large European cohort.

Although the occurrence of gastric cancer in first-degree relatives is known to be associated with a twofold to tenfold increase in the individual risk of gastric cancer development [13, 18], molecular mechanisms for this familial aggregation are unclear and it is surmised that shared environmental factors like diet and *Helicobacter pylori* infections might play key roles. Recently, a group of researchers from South Korea found that eradication of *Helicobacter pylori* in first-degree relatives of gastric cancer patients can reduce the cancer risk (1.2% vs. 2.7% in the treatment and placebo groups, respectively) [19]; however, the prevalence of *Helicobacter pylori* is low in most high-income countries [20], which is also reflected in our cohort with 29% positivity in the overall cohort versus 41% in the subgroup of Asian descent. Thus, this risk factor in western cohorts might not be of the same importance for higher incidence rates. In our analysis, potential environmental factors including *Helicobacter* infection, nicotine abuse, alcohol consumption, and higher BMI were not associated with a family history of gastroesophageal cancer. Thus, in western cohorts, genetic syndromes might be a more important player than environmental factors.

Consequently, genetic testing, although not recommended for the general gastroesophageal cancer population, might improve patient management [6, 7]; however, genetic counselling is often accompanied by reluctance from patients and healthcare professionals [4].

On one hand, skepticism about genetic testing is founded in historical events and fuelled by populist ideologies [21, 22]. On the other hand, uncertainty about legislation as well as incomprehensible laws about genetic testing, might complicate the process of genetic counselling [23]. In consequence, patients as well as treating physicians are often unwilling to talk about genetic testing. Thus, it is of utmost importance to identify subgroups who benefit most from genetic counselling.

In order to put more emphasis on preventative and early diagnostic procedures for high-risk individuals, concrete data from large cohorts such as ours are essential. Our analysis provides the insight that a positive family history is frequent in patients with gastroesophageal cancer. Especially in female patients, who experience gastroesophageal cancer less often than males, high percentages of a positive family history of cancer in general as well as gastroesophageal cancer were notable. This is in line with previous findings that female patients with a positive family history are more susceptible to developing gastric cancer, which was in part associated with genetic factors having a stronger influence on the female gender [24, 25].

Furthermore, it has been reported that the number and the degree of relatives affected by gastric cancer are associated with higher risk of developing the disease [26]; however, we could not observe a significant impact on survival concerning the degree and the number of relatives.

Yet, patients with a positive family history in general were statistically significantly diagnosed in earlier stages in our cohort. Contrary to data from Asian cohorts, we could not show that family history was associated with better prognosis in more advanced tumor stages [27], but that the stage itself remains the most prognostic factor in gastroesophageal tumors in our analysis. Thus, it is of utmost importance that patients are diagnosed early. Especially in older patients, who are known to have worse prognosis, a positive family history was associated with prolonged OS [28], while in patients with a negative family history of gastroesophageal cancer, younger patients had favorable outcomes. Thus, more efficient surveillance of patients with a positive family history might lead to beneficial outcomes independent of patient age; however, prospective data in western cohorts are missing and retrospective analyses like ours pose some disadvantages. Therefore, some limitations need to be addressed. Although reporting of family history was gathered in a structured manner, missing data due to imprecise self-reporting poses a potential bias. Furthermore, information on genetic testing is not centrally stored due to strict documentation policies, which do not allow the evaluation of any associations between hereditary and familial cancer. Thus, large-scal prospective studies with modern cohorts are warranted to evaluate the cost-effectiveness of genetic testing and regular endoscopy in patients with a positive family history [16].

In conclusion, our analysis offers a valuable contribution to the research of family history in western gastroesophageal cancer cohorts. Based on our results, we demonstrate that patients with a positive family history have distinct clinical features and support that specific screening and surveillance strategies including genetic testing and regular endoscopy should be evaluated in individuals with a positive family history in large prospective trials.

## Supplementary Information

Supplementary figure 1: Distribution of positive family history of cancer in general (A) and for gastroesophageal cancer (B) in different age groups.

Supplementary figure 2: Overall survival in adenocarcinoma patients with positive family history in general (A) and gastroesophageal cancer (B) as well as in squamous cell carcinoma patients with positive family history in general (C) and gastroesophageal cancer (D)

Supplementary table 1: Patient characteristics and their association with the overall survival (log rank test).

Supplementary table 2: Tumor characteristics and their association with the overall survival (log rank test).

Supplementary table 3: Symptoms and their association with the overall survival (log rank test).

Supplementary table 4: Therapeutic strategies. neoadjuvant regimen given to oligometastatic patients in order to achieve surgical respectability. *560 (79%) of patients received first line chemotherapy without prior systemic therapy for gastroesophageal cancer, **102 (38%) received definitive chemoradiotherapy.

Supplementary table 5: Multivariable Cox regression analysis for positive family history of cancer in general.

Supplementary table 6: Multivariable Cox regression analysis for positive family history of gastroesophageal cancer.

## Data Availability

The data that support the findings of this study are available from the corresponding author upon reasonable request.

## Abbreviations

AC: Adenocarcinoma
BMI: Body mass index
CI: Confidence interval
CPS: Combined positive score
dMMR: Mismatch repair deficiency
GEJ: Gastroesophageal junction
HER2: Human epidermal growth factor receptor 2
HR: Hazard ratio
OS: Overall survival
PD-L1: Programmed cell death ligand 1
SCC: Squamous cell carcinoma
TPS: Tumor proportion score
