# Correction to: Association of family history with patient characteristics and prognosis in a large European gastroesophageal cancer cohort

**DOI:** 10.1007/s00508-024-02456-9

**Published:** 2024-10-18

**Authors:** Hannah C. Puhr, Luzia Berchtold, Linda Zingerle, Melanie Felfernig, Lisa Weissenbacher, Gerd Jomrich, Reza Asari, Sebastian F. Schoppmann, Gerald W. Prager, Elisabeth S. Bergen, Anna S. Berghoff, Matthias Preusser, Aysegül Ilhan-Mutlu

**Affiliations:** 1 Department of Medicine I, Division of Oncology, Medical University of Vienna, Waehringer Guertel 18–20, 1090 Vienna, Austria; 2 Institute for Medical Statistics, Center for Medical Data Science, Medical University of Vienna, Vienna, Austria; 3 Department of Surgery, Medical University of Vienna, Waehringer Guertel 18–20, 1090 Vienna, Austria

**Correction to:**

**Wien Klin Wochenschr 2024**

10.1007/s00508-024-02432-3

In this article the author sequence is incorrectly given as:

Aysegül Ilhan-Mutlu, Hannah C. Puhr, Luzia Berchtold, Linda Zingerle, Melanie Felfernig, Lisa Weissenbacher, Gerd Jomrich, Reza Asari, Sebastian F. Schoppmann, Gerald W. Prager, Elisabeth S. Bergen, Anna S. Berghoff, Matthias Preusser.

It should have been:

Hannah C. Puhr, Luzia Berchtold, Linda Zingerle, Melanie Felfernig, Lisa Weissenbacher, Gerd Jomrich, Reza Asari, Sebastian F. Schoppmann, Gerald W. Prager, Elisabeth S. Bergen, Anna S. Berghoff, Matthias Preusser, Aysegül Ilhan-Mutlu.

The original article has been corrected.

