# Supplementary figures and images for: Association of family history with patient characteristics and prognosis in a large European gastroesophageal cancer cohort
Source: Wien Klin Wochenschr. 2024 Sep 5;137(7-8):214–23. doi: 10.1007/s00508-024-02432-3 (PMC12006227; doi:10.1007/s00508-024-02432-3)

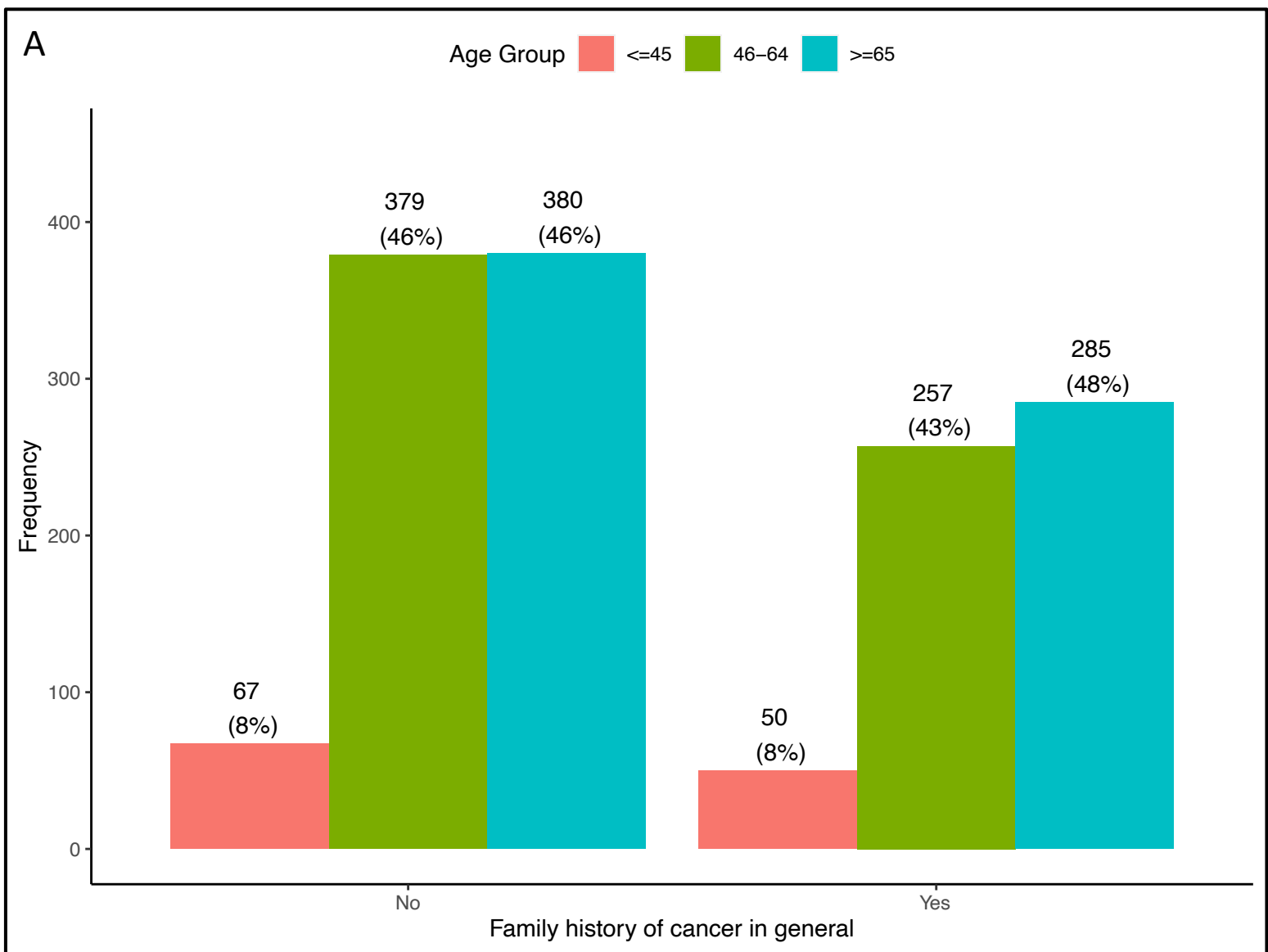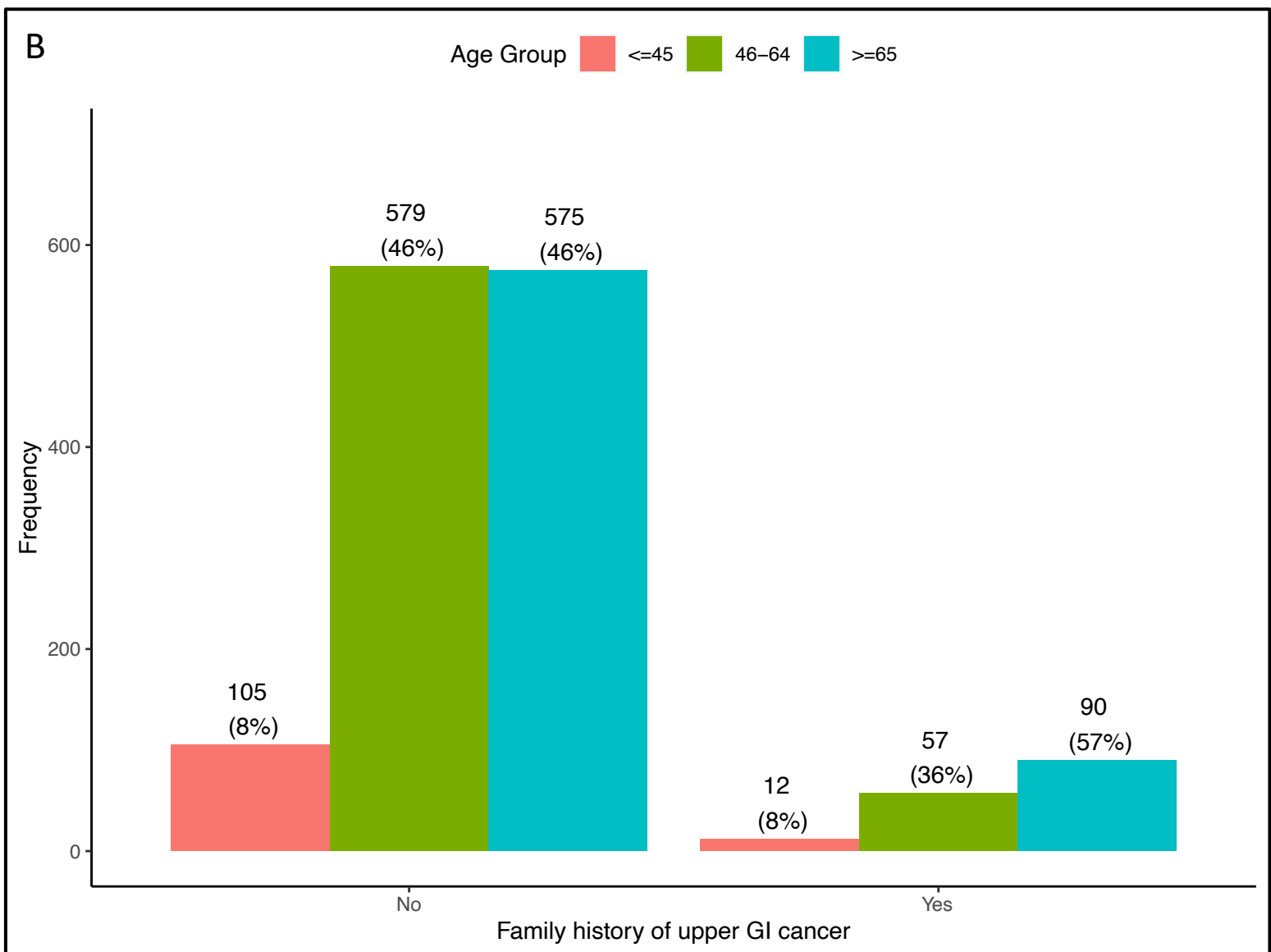

Supplement: Supplementary file 1 — Supplementary figure 1: Distribution of positive family history of cancer in general (A) and for gastroesophageal cancer (B) in different age groups. [file 508_2024_2432_MOESM1_ESM.pdf]

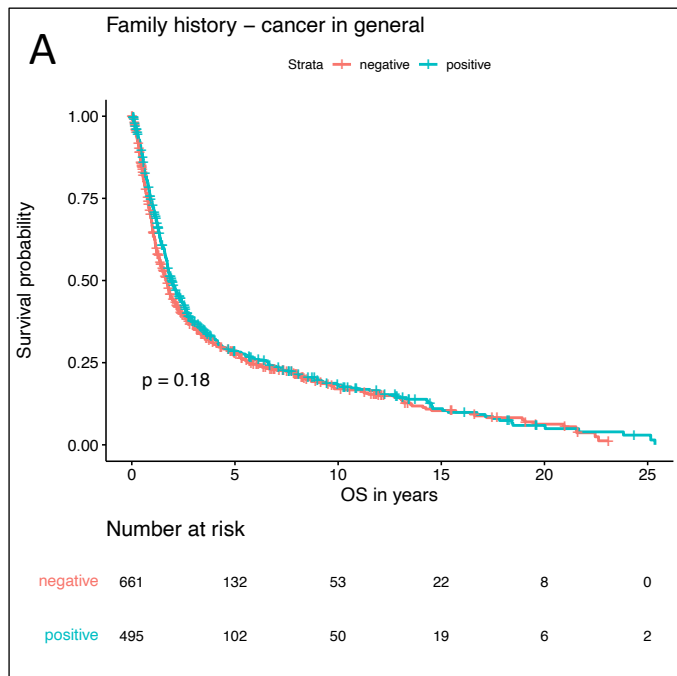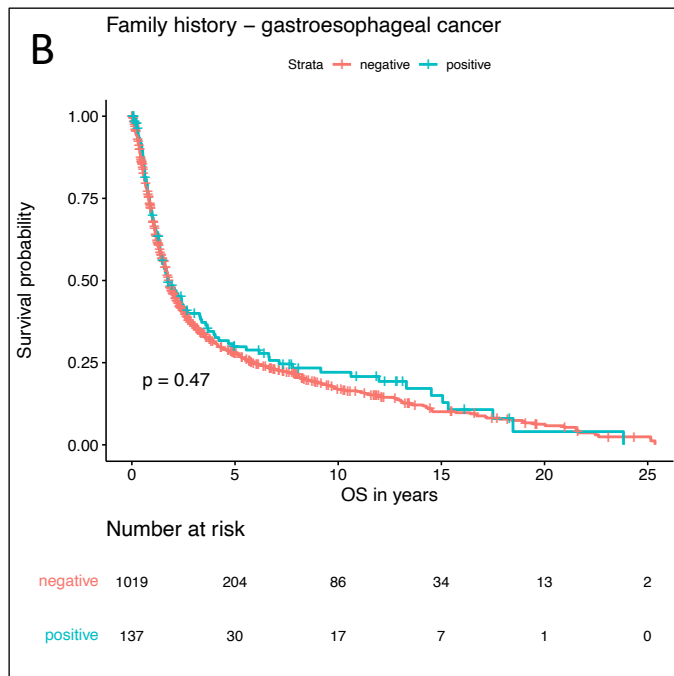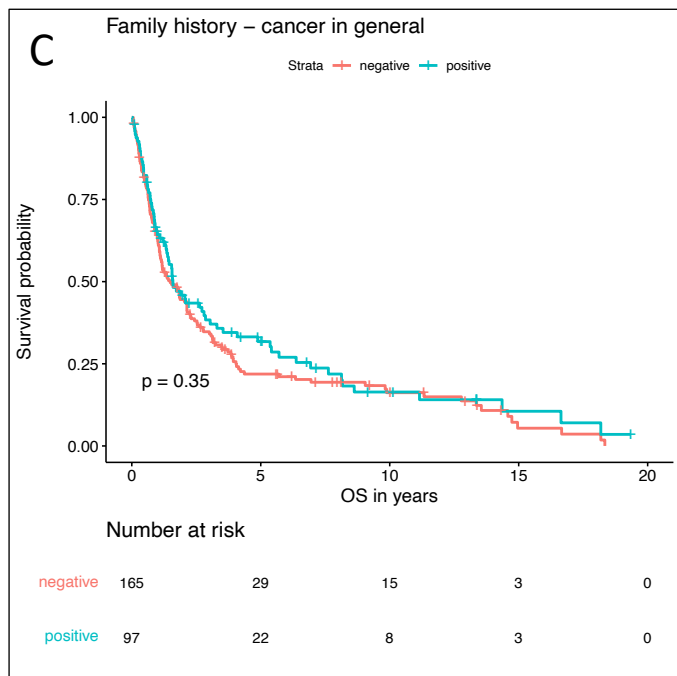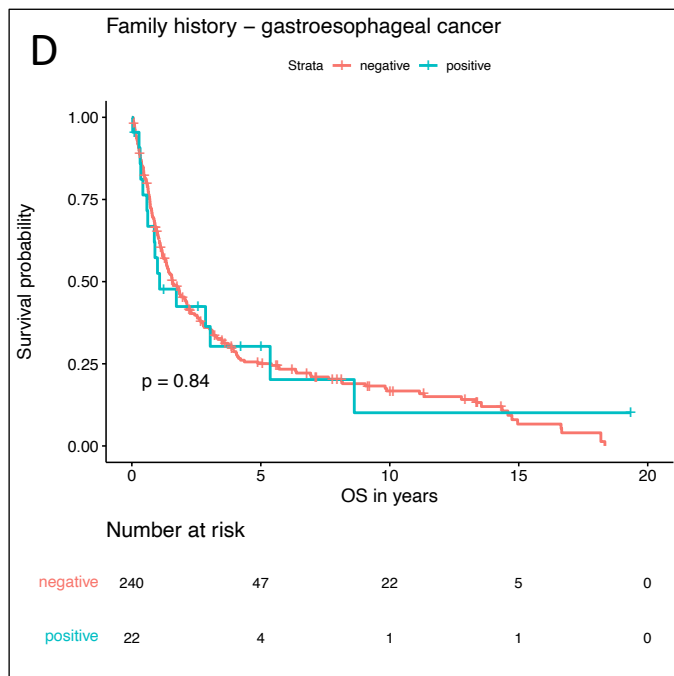

Supplement: Supplementary file 2 — Supplementary figure 2: Overall survival in adenocarcinoma patients with positive family history in general (A) and gastroesophageal cancer (B) as well as in squamous cell carcinoma patients with positive family history in general (C) and gastroesophageal cancer (D) [file 508_2024_2432_MOESM2_ESM.pdf]
