# Supplementary material for: Association of family history with patient characteristics and prognosis in a large European gastroesophageal cancer cohort
Source: Wien Klin Wochenschr. 2024 Sep 5;137(7-8):214–23. doi: 10.1007/s00508-024-02432-3 (PMC12006227; doi:10.1007/s00508-024-02432-3)
Supplement: Supplementary file 3 — Supplementary table 1: Patient characteristics and their association with the overall survival (log rank test). [file 508_2024_2432_MOESM3_ESM.docx]

| **Characteristics** | **Value, n (%)** | **Median OS in months (95%CI)** | **p-value** |
| --- | --- | --- | --- |
| **Sex** |  |  | p=0.91 |
| male | 1235 ( 70 %) | 21.7 (20.0-23.2) |  |
| female | 527 ( 30 %) | 21.5 (19.5-25.1) |  |
| **Age*** |  |  | **p=0.0023** |
| ≤45 | 132 ( 7 %) | 26.2 (21.5-33.3) |  |
| 46-64 | 780 ( 44 %) | 21.6 (20.1-24.9) |  |
| ≥65 | 850 ( 48 %) | 20.8 (18.1-22.7) |  |
| **Year of first diagnosis** |  |  | **p < 0.0001** |
| 1990-1995 | 113 ( 6 %) | 29.2 (17.8-49.5) |  |
| 1996-2000 | 203 ( 12 %) | 24.1 (19.5-30.9) |  |
| 2001-2005 | 253 ( 14 %) | 31.2 (26.2-39.3) |  |
| 2006-2010 | 434 ( 25 %) | 16.5 (14.4-19.5) |  |
| 2011-2015 | 319 ( 18 %) | 21.8 (19.6-26.1) |  |
| 2016-2023 | 440 ( 25 %) | 21.2 (17.4-24.0) |  |
| **Body mass index**** |  |  | **p=0.00014** |
| Underweight | 68 ( 5 %) | 13.3 (7.7-16.4) |  |
| Normal weight | 648 ( 50 %) | 20.9 (18.6-24.5) |  |
| Overweight | 402 ( 31 %) | 23.3 (20.7-26.6) |  |
| Obese | 169 ( 13 %) | 23.1 (18.0-40.6) |  |
| Missing data | 475 |  |  |
| **Alcohol** |  |  | **p=0.0099** |
| No alcohol | 686 ( 45 %) | 21.5 (19.4-24.8) |  |
| Moderate | 620 ( 41 %) | 22.2 (20.8-26.3) |  |
| Abuse | 214 ( 14 %) | 16.8 (14.2-22.8) |  |
| Missing data | 242 |  |  |
| **Nicotine** |  |  | p=0.19 |
| No nicotine abuse | 642 ( 42 %) | 20.7 (18.0-22.2) |  |
| Nicotine abuse | 893 ( 58 %) | 22.2 (20.9-26.0) |  |
| Missing data | 227 |  |  |

Supplementary table 1: Patient characteristics and their association with the overall survival (log rank test).
* Age was also associated with the OS as continuous variable in cox regression model: HR 1.01 (95%CI 1.01-1.02), p<0.001

** BMI was also associated with the OS as continuous variable in cox regression model: HR 0.98 (95%CI 0.96-0.99), p=0.002
