# Supplementary material for: Association of family history with patient characteristics and prognosis in a large European gastroesophageal cancer cohort
Source: Wien Klin Wochenschr. 2024 Sep 5;137(7-8):214–23. doi: 10.1007/s00508-024-02432-3 (PMC12006227; doi:10.1007/s00508-024-02432-3)
Supplement: Supplementary file 4 — Supplementary table 2: Tumor characteristics and their association with the overall survival (log rank test). [file 508_2024_2432_MOESM4_ESM.docx]

| **Characteristics** | **Value, n (%)** | **median OS in months (95%CI)** | **p-value** |
| --- | --- | --- | --- |
| **Histological subtype** |  |  | **p=0.0093** |
| Adeno | 1405 ( 80 %) | 21.9 (20.9-23.8) |  |
| SCC | 357 ( 20 %) | 19.0 (16.2-23.1) |  |
| **Tumor location** |  |  | p=0.054 |
| Stomach | 696 ( 40 %) | 21.5 (20.6-24.9) |  |
| GEJ | 539 ( 30 %) | 21.8 (19.5-24.9) |  |
| Esophagus | 527 ( 30 %) | 21.2 (17.3-24.3) |  |
| **Stages** |  |  | **p<0.0001** |
| Stage 1 | 238 ( 14 %) | 91.8 (75.2-117.2) |  |
| Stage 2 | 346 ( 20 %) | 37.2 (30.9-47.2) |  |
| Stage 3 | 584 ( 33 %) | 24.3 (22.1-27.8) |  |
| Stage 4 | 594 ( 34 %) | 10.7 (9.5-11.9) |  |
| **Lauren classification** |  |  | **p=0.00015** |
| Intestinal | 370 ( 46 %) | 27.4 (22.6-38.8) |  |
| Diffuse | 395 ( 49 %) | 19.6 (17.4-21.5) |  |
| Mixed | 41 ( 5 %) | 24.9 (13.8-35.4) |  |
| Missing | 956 |  |  |
| **Signet ring cells** |  |  | p=0.065 |
| Signet ring cells | 525 ( 30 %) | 22.2 (20.9-24.9) |  |
| No signet ring cells | 1220 ( 70 %) | 20.6 (18.4-22.9) |  |
| Missing | 17 |  |  |
| **Helicobacter pylori** |  |  | **p=0.0084** |
| H. pylori infection | 503 ( 46 %) | 26.5 (22.6-32.8) |  |
| No H. pylori infection | 591 ( 54 %) | 21.9 (20.2-25.5) |  |
| Missing | 668 |  |  |
| **Mismatch repair deficiency** |  |  | p=0.36 |
| dMMR | 12 ( 5 %) | 19.4 (10.0-NA) |  |
| pMMR | 220 ( 95 %) | 26.1 (22.8-46.7) |  |
| Missing | 1530 |  |  |
| **HER2** |  |  | **p=0.044** |
| HER2 positive | 126 ( 21 %) | 30.3 (22.8-36.9) |  |
| HER2 negative | 467 ( 79 %) | 20.6 (17.2-21.8) |  |
| Missing | 1169 |  |  |
| **PD-L1** |  |  | p=0.13 |
| PDL1 positive | 120 ( 67 %) | 22.8 (17.6-42.4) |  |
| PDL1 negative | 59 ( 33 %) | 20.5 (14.5-25.5) |  |
| Missing | 1583 |  |  |

Supplementary table 2: Tumor characteristics and their association with the overall survival (log rank test).
