# Supplementary material for: Association of family history with patient characteristics and prognosis in a large European gastroesophageal cancer cohort
Source: Wien Klin Wochenschr. 2024 Sep 5;137(7-8):214–23. doi: 10.1007/s00508-024-02432-3 (PMC12006227; doi:10.1007/s00508-024-02432-3)
Supplement: Supplementary file 5 — Supplementary table 3: Symptoms and their association with the overall survival (log rank test). [file 508_2024_2432_MOESM5_ESM.docx]

| **Characteristics** | **Value, n (%)** | **Median OS in months (95%CI)** | **p-value** |
| --- | --- | --- | --- |
| **Dysphagia** |  |  | **p=0.0012** |
| No | 666 ( 41 %) | 23.3 20.9 28.8 |  |
| Yes | 960 ( 59 %) | 20.6 18.0 22.0 |  |
| Missing | 136 |  |  |
| **Dyspepsia** |  |  | **p=0.0034** |
| No | 364 ( 22 %) | 19.1 16.7 23.5 |  |
| Yes | 1283 ( 78%) | 22.0 20.9 24.5 |  |
| Missing | 115 |  |  |
| **Acid reflux** |  |  | p=0.95 |
| No | 1275 ( 85 %) | 21.9 20.9 24.1 |  |
| Yes | 221 ( 15 %) | 20.8 17.0 26.7 |  |
| Missing | 266 |  |  |
| **Abdominal pain** |  |  | p=0.72 |
| No | 1118 ( 75 %) | 22.0 20.9 24.5 |  |
| Yes | 377 ( 25%) | 20.9 18.0 26.2 |  |
| Missing | 267 |  |  |
| **Nausea** |  |  | p=0.071 |
| No | 1289 ( 86 %) | 22.5 21.2 25.4 |  |
| Yes | 207 ( 14 %) | 16.5 13.4 20.6 |  |
| Missing | 266 |  |  |
| **GI bleeding** |  |  | **p=0.015** |
| No | 1243 ( 77 %) | 20.6 19.1 22.0 |  |
| Yes – ulceration | 141 ( 9 %) | 19.4 16.6 28.0 |  |
| Yes – active bleeding | 225 ( 14 %) | 23.8 20.9 33.3 |  |
| Missing | 153 |  |  |
| **Frailty** |  |  | **p<0.0001** |
| No | 1340 ( 83 %) | 22.5 21.0 25.2 |  |
| Yes | 266 ( 17 %) | 13.0 11.5 17.3 |  |
| Missing | 156 |  |  |
| **Weight loss** |  |  | **p<0.0001** |
| No | 805 ( 50 %) | 26.1 23.1 30.7 |  |
| Yes | 800 ( 50 %) | 16.2 14.6 18.0 |  |
| Missing | 157 |  |  |

Supplementary table 3: Symptoms and their association with the overall survival (log rank test).
