# Supplementary material for: Association of family history with patient characteristics and prognosis in a large European gastroesophageal cancer cohort
Source: Wien Klin Wochenschr. 2024 Sep 5;137(7-8):214–23. doi: 10.1007/s00508-024-02432-3 (PMC12006227; doi:10.1007/s00508-024-02432-3)
Supplement: Supplementary file 6 — Supplementary table 4: Therapeutic strategies. neoadjuvant regimen given to oligometastatic patients in order to achieve surgical respectability. *560 (79%) of patients received first line chemotherapy without prior systemic therapy for gastroesophageal cancer, **102 (38%) received definitive chemoradiotherapy. [file 508_2024_2432_MOESM6_ESM.docx]

| Characteristics | Value, n (%) |
| --- | --- |
| Surgery |  |
| Surgical resection | 1201 ( 68 %) |
| Systemic therapy |  |
| Neoadjuvant/perioperative | 368 ( 21 %) |
| Pseudoneoadjuvant^ | 13 ( 1 %) |
| Adjuvant (without neoadjuvant regimen) | 127 ( 7 %) |
| Palliative with initial curative intent | 63 ( 4 %) |
| Palliative 1^st^ line | 646 ( 37 %)* |
| Palliative 2^nd^ line | 274 ( 16 %) |
| Palliative 3^rd^ line | 96 ( 5 %) |
| Palliative 4^th^ line | 22 ( 1 %) |
| Palliative 5^th^ line | 10 ( 1 %) |
| Radiotherapy |  |
| Radiotherapy of primary tumor | 269 ( 15 %)** |
| Radiotherapy of primary tumor recurrence | 56 ( 3 %) |
| Radiotherapy of metastatic sites | 119 ( 7 %) |

Supplementary table 4: Therapeutic strategies
^neoadjuvant regimen given to oligometastatic patients in order to achieve surgical respectability.
*560 (79%) of patients received first line chemotherapy without prior systemic therapy for gastroesophageal cancer, **102 (38%) received definitive radiochemotherapy
