# Supplementary material for: Association of family history with patient characteristics and prognosis in a large European gastroesophageal cancer cohort
Source: Wien Klin Wochenschr. 2024 Sep 5;137(7-8):214–23. doi: 10.1007/s00508-024-02432-3 (PMC12006227; doi:10.1007/s00508-024-02432-3)
Supplement: Supplementary file 7 — Supplementary table 5: Multivariable Cox regression analysis for positive family history of cancer in general. [file 508_2024_2432_MOESM7_ESM.docx]

|  |  | Univariable analysis | | | | Multivariable analysis | | | | |
| --- | --- | --- | --- | --- | --- | --- | --- | --- | --- | --- |
| Characteristic | N | **HR1** | **95% CI1** | **p-value** | | HR1 | 95% CI1 | | p-value | |
| **Family history in general** | 1,126 | 0.9 | 0.8, 1.02 | 0.1 | | 1.37 | 1.11, 1.69 | | **0.004** | |
| **BMI** | 1,126 | 0.98 | 0.96, 0.99 | **0.002** | | 0.98 | 0.97, 1.00 | | 0.069 | |
| **Stages** |  |  |  |  | |  |  | |  | |
| Stage 1 | 156 | — | — |  | | — | — | |  | |
| Stage 2 | 192 | 1.75 | 1.42, 2.15 | **<0.001** | | 1.99 | 1.52, 2.61 | | **<0.001** | |
| Stage 3 | 374 | 2.2 | 1.81, 2.67 | **<0.001** | | 2.31 | 1.79, 2.99 | | **<0.001** | |
| Stage 4 | 404 | 5.46 | 4.49 6.64 | **<0.001** | | 7.04 | 5.46, 9.06 | | **<0.001** | |
| **Alcohol consumption** |  |  |  |  | |  |  | |  | |
| No alcohol | 494 | — | — |  | | — | — | |  | |
| Moderate | 481 | 0.92 | 0.81, 1.04 | 0.2 | | 0.88 | 0.76, 1.02 | | 0.10 | |
| Abuse | 151 | 1.2 | 1.01, 1.43 | **0.034** | | 1.13 | 0.91, 1.40 | | 0.3 | |
| **Age category** |  |  |  |  | |  |  | |  | |
| >45&<65 | 489 | — | — |  | | — | — | |  | |
| <=45 | 86 | 1.18 | 0.94, 1.47 | 0.2 | | 0.88 | 0.60, 1.28 | | 0.5 | |
| >=65 | 551 | 1.37 | 1.10, 1.71 | **0.005** | | 1.53 | 1.27, 1.84 | | **<0.001** | |
| **Tumor location** |  |  |  |  | |  |  | |  | |
| GEJ | 383 | — | — |  | | — | — | |  | |
| Stomach | 427 | 1.02 | 0.9, 1.16 | 0.8 | | 1.02 | 0.86, 1.21 | | 0.8 | |
| Esphagus | 316 | 1.16 | 1.02, 1.34 | **0.029** | | 1.27 | 1.03, 1.57 | | **0.025** | |
| **Histological subtype** |  |  |  |  | |  |  | |  | |
| AC | 927 | — | — |  | | — | — | |  | |
| SSC | 199 | 1.19 | 1.04, 1.35 | **0.009** | | 0.94 | 0.75, 1.19 | | 0.6 | |
| **Family history in general ***  **Age category** | 1,126 |  |  |  | |  |  | |  | |
| Family history in general * <=45 | 86 |  |  |  | | 0.61 | 0.34, 1.11 | | 0.10 | |
| Family history in general * >=65 | 551 |  |  |  | | 0.65 | 0.49, 0.86 | | **0.003** | |
| 1HR = Hazard Ratio, CI = Confidence Interval | | | | |  | | |  | |  |

Supplementary table 5: Multivariable Cox regression analysis for positive family history for cancer in general
